# Association noma aigu – VIH – malnutrition sévère chez l'enfant: à propos de 2 cas

**Published:** 2012-09-01

**Authors:** Toni Kasole Lubala, Augustin Mulangu Mutombo, Kabiriko Olivier Mukuku, Makinko Paul Ilunga, Mick Pongombo Shongoya

**Affiliations:** 1 Service de Pédiatrie Générale de l'Hôpital Général de Référence Jason Sendwe de Lubumbashi, RDC

**Keywords:** Noma, VIH, enfant, malnutrition, Lubumbashi, Noma, HIV, child, malnutrition, Lubumbashi

## Abstract

Les auteurs rapportent deux cas de Noma aigu. Ľun ďeux ayant une présentation clinique et topographique ďun noma neonatorum observé chez un enfant congolais de 3 ans, VIH séropositif au stade clinique et biologique IV (malnutrition sévère et taux de CD4 à 6%). Les lésions auraient été favorisées par une gingivo-stomatite à Candida albicans. Ľécouvillonnage naso-pharyngé réalisé après le début de ľantibiothérapie n'a pas mis en évidence de germe. Dans le cas numéro 2, la progression des lésions a pu être maitrisée grâce à une antibiothérapie à large spectre faite de cefotaxime et métronidazole. Une prise en charge nutritionnelle au F75 puis F100 a été administrée avec succès. Dans le premier cas, le patient est décédé.

## Introduction

Le Noma, également connu sous le nom de «cancrumoris» est une affection mutilante connue depuis ľantiquité et désignée sous ce nom par Lund en 1762 [1]. Elle sévit surtout chez l'enfant entre 1 à 4 ans dans les pays en développement. Elle est de ce fait considérée par l'OMS comme une priorité de sante publique. Dans certaines régions d'Afrique son incidence varie de 2 à 4 cas pour 10.000 enfants [1]. Il résulte d'une interaction complexe entre de nombreux facteurs dont les plus cités sont l'infection, l'immunodépression et la malnutrition [2]. L'infection par le VIH accroit le risque d'apparition de la maladie du fait de l'immunodépression et des infections opportunistes qu'elle favorise. En effet, des études étiologiques rapportent l'implication de bactéries anaérobies strictes, notamment le *Fusobacterium nécrophorum* [2]. Nous rapportons ici deux cas de Noma survenus chez des patients de 19 mois et de 3 ans infectés par le VIH et malnutris sévères, hospitalisés en décembre 2010 à l'hôpital Général de référence Jason Sendwe de Lubumbashi en République Démocratique du Congo.

## Patients et observations

### Observation 1

L'enfant B.B. de sexe féminin âgée de 19 mois pesant 7 kg a été transférée de Kolwezi à l'hôpital Général de Référence Sendwe, pour une ulcération prenant l'hémiface gauche. Elle est née à terme, d'un accouchement eutocique, avec poids à la naissance 2500grammes. Le calendrier vaccinal est bien respecté (BCG, DTCQ, POLIO et ROUGEOLE) et son alimentation est mixte. A l'interrogatoire, la mère nous a révélée qu'elle a eu des rapports sexuels occasionnels avec plusieurs partenaires, elle a perdu un enfant 4 ans auparavant.

**Figure 1:**
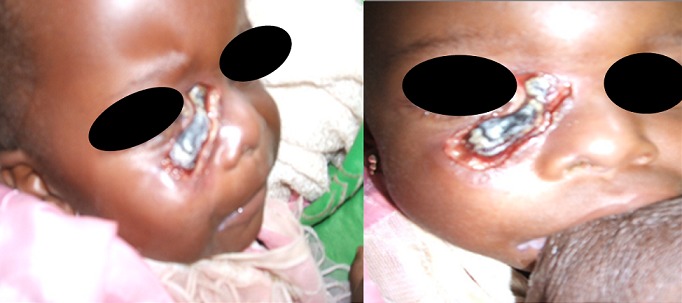
Fillette de 19 mois présentant un Noma facial aigu et une malnutrition sévère associée à une infection à VIH

Le début de la maladie a été brutal et marquée par la fièvre, des pleurs incessants, l'anorexie et l'altération de l'état général avec les signes d'anémie notamment pâleur palmaire et asthénie. Dans l'évolution, nous avons noté une altération sévère de l'état général avec des signes de malnutrition au stade sévère (Z-score poids pour âge=-3,38 ET), une apparition d'un sillon délimitant une zone nécrotique centrale allant de l'angle interne de l'œil droit s'étendant jusqu'à la joue du même côté, une perte de substance rapide et importante et un œdème facial (Figure 1). L'extension rapide de la nécrose initialement occupant l'espace interoculaire jusqu'au niveau de la mâchoire supérieure en moins d'un mois nous a poussé à pratiquer une sérologie VIH par le détermine, unigold, double check positif, l'Elisa positif, le taux de CD4 à 10% et la PCR RNA positive.

Nous avons conclu à un Noma aigu survenu sur un terrain d'immunodépression sévère à VIH avec malnutrition sévère. Nous avons hospitalisé cet enfant dans l'unité de nutrition thérapeutique intensive de l'hôpital. Sept jours après le début du traitement, l'enfant est décédé dans un tableau de détresse respiratoire aigüe avec anémie sévère.

### Observation 2

L'enfant L.M. âgé de 3 ans, de sexe masculin est admis aux urgences de l'hôpital Provincial de référence Jason Sendwe pour ulcérations et perte de substance cutanée au niveau du nez, de la face externe de la cuisse droite ainsi que de la marge anale dans un contexte fébrile.

A l'anamnèse, les parents décrivent une installation progressive ayant débuté par des phlyctènes qui ont fistulisé, laissant place à des lésions ulcéro-nécrosantes. Le niveau socio-économique de la famille semble précaire. L'enfant est cadet d'une fratrie de 9 dont 3 décès à la suite d'épisodes infectieux non déterminés. Les deux parents sont en vie et en bonne santé apparente. Le patient a été nourris exclusivement au sein jusqu'à 12 mois.

**Figure 2:**
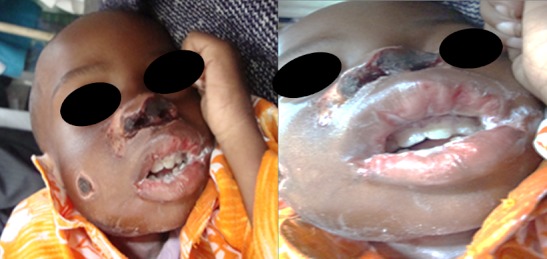
Garcon de 3 ans présentant un noma facial aigu, une malnutrition sévère et une infection à VIH: Les zones de nécrose tissulaire et la candidose buccale sont bien visibles

**Figure 3:**
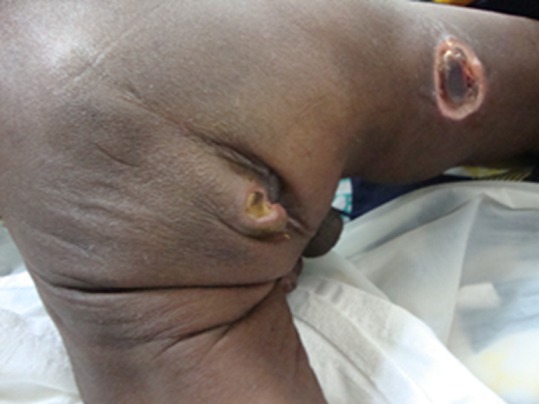
Les lésions ulcéro-nécrotiques au niveau de la marge anale sont bien visibles

A l'examen clinique, le patient présente un état général altéré, il est extrêmement amaigri. Il a un poids de 6,8kg (inférieur au percentile 3), une taille de 72 cm (inférieur au percentile 3), et un périmètre brachial inférieur à 12 cm. Ses indices anthropométriques calculés par le Z-score sont les suivants : Z-score poids pour âge est de -5,549, Z-score poids pour taille est de -3,550 et Z-score taille pour âge est de -6,498. Son haleine est fétide. Il présente des lésions ulcéro-nécrotiques au niveau de la lèvre supérieure et du nez (Figure 2). Des lésions de même nature sont observées au niveau de la marge anale et de la face externe de la cuisse droite (Figure 3). L'examen endobuccal, difficile à cause de la contracture des muscles de la mâchoire, révèle en outre une gingivo-stomatite étendue avec un muguet diffus saignant au raclage évoquant une candidose buccale étendue.

Le bilan biologique à l'admission révèle une hyperleucocytose (24000/mm^3^) et une formule neutrophilique à 80%. La vitesse de sédimentation est très élevée (122 millimètres à la 1ère heure). Il y a présence de l'anticorps anti-VIH. Le pourcentage lymphocytaire de CD4 est à 6%, suggérant une classification biologique au stade IV. Pas de germe isolé à l'écouvillonnage bucco-pharyngé. Le tableau clinique est compatible avec un Noma à la phase aigüe de son évolution associé à une infection à VIH au stade clinique et biologique IV.

**Figure 4:**
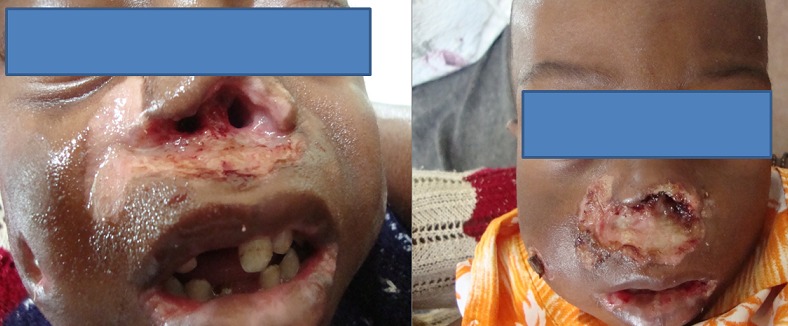
Chute du tissus nécrotique et contrôle de la candidose buccale obtenus au 12e jour

**Figure 5:**
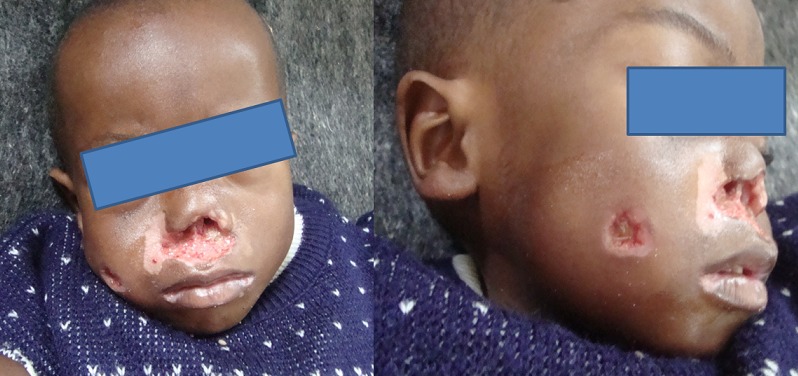
Le début du processus de cicatrisation est bien visible. Photo prise au 14e jour

Une antibiothérapie faite de céfotaxime (100mg/kg/j) et Métronidazole (30 mg/kg/j) a été administrée pendant 15 jours associée à un traitement antimycosique topique fait de nystatine en guise de traitement de la gingivo-stomatite mycosique. Une nutrition thérapeutique a été conduite dans le même temps avec du lait thérapeutique F75 en 1 ^ère^ phase, puis F100 en 2^ème^ phase. La chute des tissus nécrotiques intervient au 12^ème^ jour de traitement (Figure 4). Son évolution était bonne et marquée par un début de processus de cicatrisation au 14^ème^ jour (Figure 5) et sa sortie de l'hôpital est autorisée au 20^ème^ jour.

## Discussion

Dans nos observations, les patients ont développé leur symptomatologie à l'âge de 19 mois et 3 ans, ce qui correspond à la tranche d'âge de forte incidence chez l'enfant [1, 3]. Cependant, la présentation topographique observée dans le cas clinique numéro 2, impliquant la face et la région ano-rectale n'est pas la forme classique décrite dans le noma. Par la topographie des lésions, notre cas se rapproche plus du noma neonatorum caractérisé par un processus de nécrose cutanéo-muqueuse de la face, mais aussi de la région ano-scrotale [4]. Par ailleurs, comme le suggère son nom, le noma neonatorum ne frappe que les nouveau-nés, plus souvent prématurés, alors que le noma concerne essentiellement la tranche d'âge de 2 à 6 ans [5]. L'importance de la perte de substance varie d'une petite surface a une nécrose plus étendue détruisant le nez, la lèvre supérieure, le maxillaire supérieur voire la marge sous orbitaire [1].

L'hyperleucocytose rapportée dans notre 2^ème^ cas a également été observée par d'autres auteurs. Elle se situe classiquement à la phase aigüe entre 20–30×10^9^/l [1, 2]. Huit cas de noma ont été décrits chez des patients VIH positifs, au Zimbabwe, en 1996; d'autres cas ont été rapportés chez des enfants en Zambie, en 1998 [5, 6]. Dans notre cas, les patients étaient séropositifs VIH1 et la maladie s'est installée chez des patients au stade d'immunodépression sévère.

Nos patientsprésentaientpar ailleurs une malnutrition sévère. Classiquement, le Noma survient surtout chez des enfants vivant dans des conditions socioéconomiques précaires, exposés à des déficiences nutritionnelles chroniques [7, 8]. La prise en charge consiste essentiellement en une prise en charge nutritionnelle, un traitement local des lésions ulcéro-nécrotiques, ainsi qu'une antibiothérapie par voie générale active sur les anaérobies [1]. Dans notre 2^ème^cas, nous avons obtenu une excellente réponse sous céfotaxime et métronidazole IV.

## Conclusion

Le Noma ne fait pas parti des affections spécifiques au VIH, ni des affections fréquentes au cours d'une infection à VIH. Pourtant dans de nombreux pays d'Afrique, de plus en plus des cas d'associations Noma-VIH sont rapportés. Associé à l'infection à VIH, le Noma semble survenir lorsque l'immunodépression est sévère. Les interactions entre le VIH/SIDA et le Noma devraient être d'avantages explorés. Une évolution favorable est possible lorsque la prise en charge est précoce
